# Supplementary material for: Polysome Profiling Proves Impaired IL-10 and Caspase-8 Translation in PBMCs of Hemodialysis Patients
Source: Biomolecules. 2025 Feb 26;15(3):335. doi: 10.3390/biom15030335 (PMC11940673; doi:10.3390/biom15030335)
Supplement: Supplementary file 1 [file biomolecules-15-00335-s001.zip › biomolecules-3450374-supplementary.pdf]

Supplementary data

Supplementary Table S1a: Fractional IL-10 translation (Ct values, basal) in HD patients stratified for the median dialysis time <66.5 and ≥66.5 months

	F5	F6	F7	F8	F9	bG
HD (<66.5)	6.4±1.5	5.6±1.2	5.5±1.2	5.4±1.6	5.1±1.0	6.4±1.2
HD (≥66.5)	6.6±3.5	5.4±2.1	5.2±1.7	5.4±1.4	5.4±1.6	6.9±1.4
ANOVA (adjusted p value)	0.999	0.999	0.999	0.999	0.999	0.999

Supplementary Table S1b: Fractional Casp-8 translation (Ct values, basal) in HD patients stratified for the median dialysis time <66.5 and ≥66.5 months

	F5	F6	F7	F8	F9	bG
HD (<66.5)	0.3±1.5	-0.74±1.0	-0.3±0.9	0.5±0.9	0.9±0.8	2.4±0.8
HD (≥66.5)	-0.2±2.1	-0.8±1.6	-0.3±1.2	0.5±0.8	1.0±0.7	2.7±0.4
ANOVA (adjusted p value)	0.999	0.999	0.999	0.999	0.999	0.999

Supplementary Table S2a: Fractional IL-10 translation (Ct values, basal) in HD patients stratified for the median kt/V value (<1.7 and ≥1.7)

	F5	F6	F7	F8	F9	bG
HD (<1.7)	6.7±3.1	5.6±1.4	5.2±1.4	5.4±1.7	5.1	6.5±1.5
HD (≥1.7)	6.3±2.2	5.4±1.9	5.4±1.5	5.6±1.4	5.4±1.4	6.8±1.2
ANOVA (adjusted p value)	0.999	0.999	0.999	0.999	0.999	0.999

Supplementary Table S2b: Fractional Casp-8 translation (Ct values, basal) in HD patients stratified for the median kt/V value (<1.7 and ≥1.7)

	F5	F6	F7	F8	F9	bG
HD (<1.7)	0.08±1.7	-0.6±1.2	-0.3±0.9	0.3±1.0	0.9±0.8	2.5±0.8
HD (≥1.7)	0.1±2.0	-0.9±1.5	-0.4±1.1	0.6±0.80	1.1±0.6	2.6±0.5
ANOVA (adjusted p value)	0.999	0.999	0.999	0.999	0.999	0.999

Supplementary Table S3a: Fractional IL-10 translation (Ct values, basal) in HD patients stratified according to permanent catheter (Pcat) or fistula (F) access

	F5	F6	F7	F8	F9	bG
Pcat	7.5±3.9	5.6±1.2	5.5±1.1	5.0±1.9	5.2±1.2	6.5±1.6
F	6.2±2.1	5.5±1.8	5.3±1.6	5.6±1.3	5.3±1.4	6.7±1.2
ANOVA (adjusted p value)	0.999	0.999	0.999	0.999	0.999	0.999

Supplementary Table S3b: Fractional Casp-8 translation (Ct values, basal) in HD patients stratified according to permanent catheter (Pcat) or fistula (F) access

	F5	F6	F7	F8	F9	bG
Pcat	0.2±2.0	-0.8±1.7	-0.2±1.1	0.08±1.2	0.9±1.0	2.3±0.8
F	0.07±1.8	-0.8±1.3	-0.4±1.0	0.6±0.7	1.0±0.6	2.7±0.6
ANOVA (adjusted p value)	0.999	0.999	0.999	0.999	0.999	0.999
